# Nationwide Medicare and Medicaid Trends in Management of Dupuytren’s Contracture

**DOI:** 10.1177/15589447251397016

**Published:** 2025-12-16

**Authors:** Evan S. Pistone, Samer W. Majeed, Grant B. Torres, Rion E. Brown, John Faillace

**Affiliations:** 1 John Sealy School of Medicine, University of Texas Medical Branch, Galveston, USA; 2 Department of Orthopedic Surgery, University of Texas Medical Branch, Galveston, USA

**Keywords:** hand, anatomy, Dupuytren’s contracture, Medicare, hand surgery, collagenase, fasciectomy

## Abstract

**Background:**

In 2020, collagenase clostridium histolyticum (CCH) was withdrawn from markets in Europe, Asia, and Australia for Dupuytren’s contracture (DC). The impact of this withdrawal on US treatment patterns remains unclear. This study evaluates national trends in DC management across Medicare and Medicaid populations.

**Methods:**

Using data from the Centers for Medicare and Medicaid Services Physician/Supplier Procedure Summary, all DC-related procedure claims from 2012 to 2022 were identified using Current Procedural Terminology (CPT) codes for open partial palmar fasciotomy (26045), open fasciectomy (26121, 26123, 26125), percutaneous needle aponeurotomy (26040), and collagenase injection (20527). Claims were analyzed by year, provider type, and place of service, normalized per 100 000 total claims.

**Results:**

Between 2012 and 2022, 189 142 procedures were recorded. Overall intervention rates increased by 36%, from 35.87 to 49.10 per 100 000 claims. Collagenase injections rose 269%, while open fasciotomy declined 26%. Open fasciectomy remained the dominant treatment, accounting for over 60% of procedures. Notably, fasciectomy with digital release (26123, 26125) increased, while isolated palmar fasciectomy (26121) decreased. Office-based procedures rose from 14% to 21%, while ambulatory surgical centers remained most common (46% in 2022). Orthopedic surgeons were the leading providers, though hand specialists saw the largest proportional increase.

**Conclusion:**

Unlike international trends, US collagenase use continued to rise through 2022. Open fasciectomy remained primary, with a shift toward minimally invasive treatments and outpatient settings. Hand specialists are increasingly involved, though orthopedic surgeons lead in volume. These findings reflect evolving care models and highlight the need for continued monitoring of provider patterns and access to DC treatment.

## Introduction

Dupuytren’s contracture (DC) is a progressive fibroproliferative disorder affecting the palmar fascia of the hand, leading to fixed flexion contractures of the digits. It is estimated to affect 3% to 6% of the Caucasian population, with higher prevalence in individuals of Northern European descent.^1,2^ The condition can significantly impact hand function and quality of life for affected individuals. In 2010, collagenase clostridium histolyticum (CCH) was approved by the US Food and Drug Administration (FDA) as a nonsurgical treatment option for DC, offering an alternative to traditional surgical approaches like fasciectomy.^3^ Collagenase clostridium histolyticum works by enzymatically breaking down the excessive collagen in the cords and nodules of DC. Its introduction was initially met with enthusiasm, as it provided a minimally invasive option that could be performed in an office setting.^4^

However, in 2020, CCH was voluntarily withdrawn by its manufacturer from the European, Asian, and Australian markets for use in DC treatment.^5^ This decision was reportedly based on commercial reasons rather than safety concerns. The removal of CCH from these major markets has raised questions about its impact on treatment patterns for DC in the United States, where it remains available.^6^ The management of DC has traditionally involved surgical interventions such as needle aponeurotomy, limited fasciectomy, and dermofasciectomy.^7^ Each of these approaches carries its own set of benefits and risks. The introduction of CCH offered a nonsurgical alternative, potentially changing the landscape of DC treatment. However, its long-term efficacy, cost-effectiveness, and impact on overall treatment patterns remain subjects of ongoing research and debate.^1,8,9^ As the prevalence of DC increases with age, it poses a significant burden on the Medicare population and the US health care system.^1,2^

Understanding the trends in DC treatment is crucial for health care policy, resource allocation, and improving patient outcomes. Changes in treatment patterns can reflect shifts in clinical practice, patient preferences, and health care economics. This study aims to investigate the broader trends and treatment-specific patterns in the management of DC within the Medicare population. By analyzing nationwide Medicare data, we seek to elucidate how the continued availability of CCH in the US market, contrasting with its withdrawal elsewhere, has influenced treatment choices. In addition, we aim to explore regional variations, provider specialties involved in DC management, and the overall trajectory of different treatment modalities over time. Our analysis will provide valuable insights into the evolving landscape of DC treatment in the United States, potentially informing clinical decision-making, health care policy, and future research directions in this field.

## Methods

This retrospective analysis was conducted using the Centers for Medicare & Medicaid Services (CMS) Physician/Supplier Procedure Summary database, a source repeatedly used to analyze procedure-specific trends.^10-12^ Deidentified patient data was extracted from the database in May of 2024 for all treatment procedure claims for DC between 2012 and 2022. Procedure type was identified using Current Procedural Terminology (CPT) codes. The procedures that were included in the study consisted of open fasciotomy (CPT 260465), open fasciectomy (CPT 26121, 26123, 26125), percutaneous needle aponeurotomy (CPT 26040), and CCH injections (CPT 20527). All patients whose procedure claim was reported in the CMS database in the 10-year period were included for analysis. Outcomes of interest were frequency of procedures, providers, and place of service.

The data obtained in our analysis were normalized to the total number of claims to the CMS database in the period studied to account for changes in the overall use of the Medicare and Medicaid services. Frequency of procedures was thus converted to a number per 100 000 Medicare and Medicaid claims. Similar methodology has been demonstrated in related papers which use the CMS database to track changes in other procedures.^13-15^

The results for the primary outcomes were organized based on provider specialty code and place of service code. Certain provider specialties were excluded from analysis due to being less than 3% of all the procedures recorded for the CPT codes in the time period. These specialties included family practice, rheumatology, general surgery, emergency medicine, ophthalmology, physical medicine and rehabilitation, general practitioner, and physician assistant. The provider specialties that were included for analysis were orthopedic surgery, hand surgeons, and plastic surgeons. Provider specialty code 49 (ambulatory surgical center) was also included as it constituted 19% of all procedures, though the exact provider who performed the procedures was unclear. Place of service codes which represented more than 3% of all procedures in the 10-year period included office, off-campus outpatient hospital, on-campus outpatient hospital, inpatient hospital, emergency room, and ambulatory surgical center.

## Results

**Figure 1.**
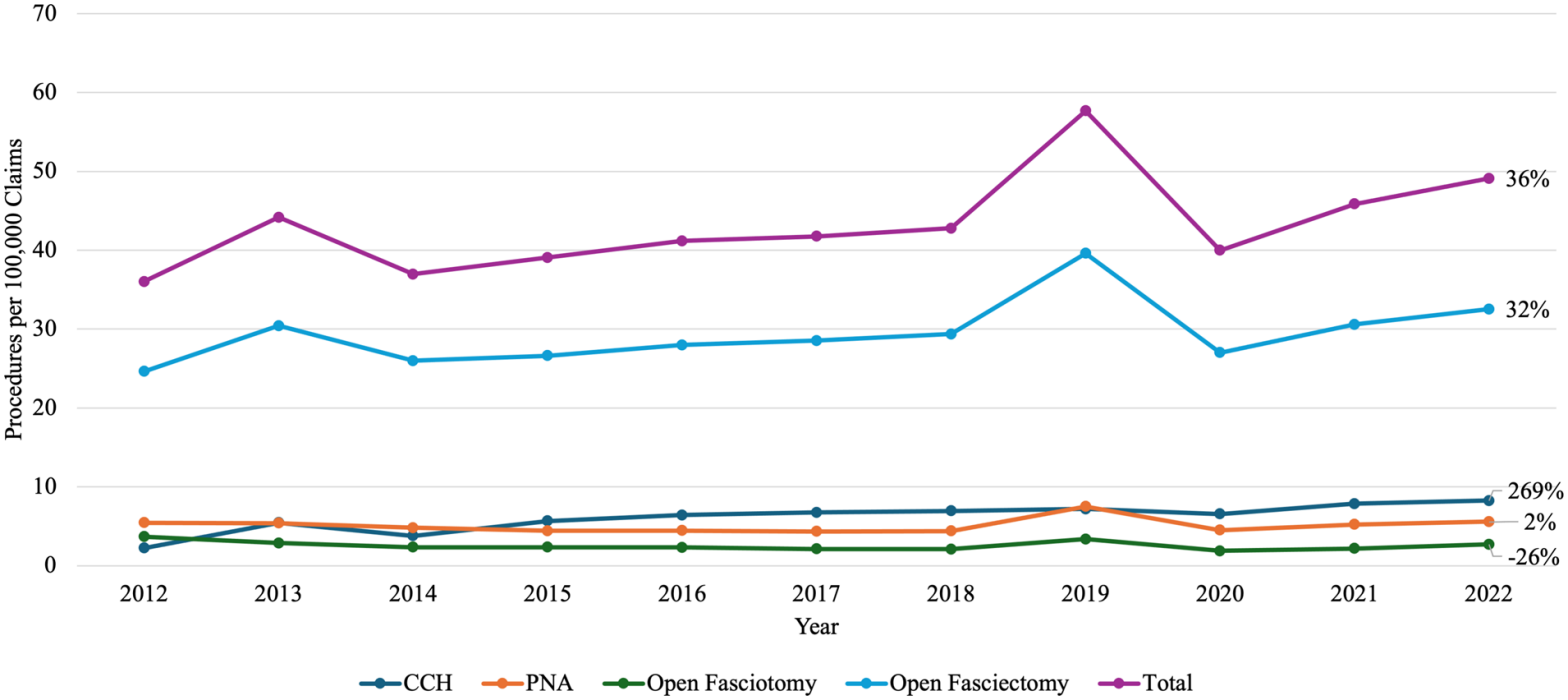
Frequency of procedures per 100 000 claims from 2012 to 2022: line graph displaying annual rates of Dupuytren’s contracture procedures per 100 000 Medicare claims from 2012 to 2022. The y-axis represents procedure rates per 100 000 claims; the x-axis shows calendar year. Lines represent trends for collagenase clostridium histolyticum (CCH), percutaneous needle aponeurotomy (PNA), open fasciotomy, open fasciectomy, and total procedural volume.

A total of 189 142 procedures between the years 2012 and 2022 were included for analysis. The frequency of each procedure is depicted in Figure 1. The frequency per 100 000 Medicare claims for all procedures measured by CPT codes increased by 36% over the 10-year period. By far the most utilized procedure was the open fasciectomy making up between 66% and 68% of the procedures performed in this cohort of CPT codes. The largest percent increase for each of the 5 procedures identified was the CCH injections, which increased by 269% from 2.24 to 8.26 procedures per 100 000 claims over the 10 years. Open fasciotomy decreased in usage by 26% from 3.68 to 2.71 procedures per 100 000 claims in the same period.

**Figure 2.**
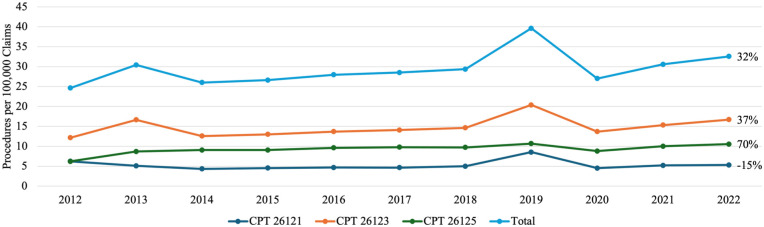
In detail frequency of open fasciectomy procedures per 100 000 claims from 2012 to 2022: line graph illustrating annual rates of open fasciectomy procedures for Dupuytren’s contracture per 100 000 Medicare claims from 2012 to 2022. The y-axis represents procedure rates per 100 000 claims; the x-axis denotes calendar years. Individual lines represent CPT codes 26121, 26123, and 26125 with an additional line depicting the combined total of all open fasciectomy procedures. CPT = Current Procedural Terminology.

Since the open fasciectomy was measured as a group of 3 CPT codes and the combined frequency of these codes was much greater than the other 4 procedure codes, more detail was needed to fully understand the trends in this procedure.^16^ Current Procedural Terminology codes 26123 and 26125 were used for open fasciectomy with digital release, while 26121 involved only the palmar fascia. Current Procedural Terminology code 26121 saw a moderate 15% decrease in usage from 6.25 to 5.31 per 100 000 claims over the 10-year period (Figure 2). Current Procedural Terminology codes 26123 and 26125 which represent open fasciectomy with digital release saw a 37% and 70% increase (Figure 2).

**Figure 3.**
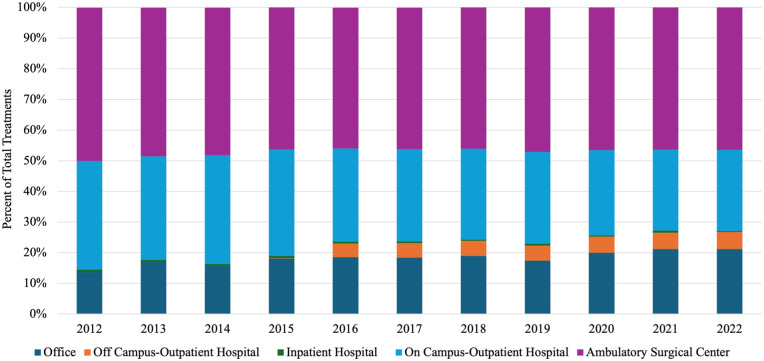
Place of service for all treatments from 2012 to 2022. Stacked bar chart showing the distribution of Dupuytren’s contracture treatments by site of service from 2012 to 2022. The y-axis indicates the percentage of total treatments, and the x-axis denotes calendar year. Bars are segmented by location of service, including office, off-campus outpatient hospital, inpatient hospital, on-campus outpatient hospital, and ambulatory surgical center.

Place of service was measured as a proportion of all total procedures for each year of the 10 years of study. Ambulatory surgical centers were the most common place of service but saw a slight decline of 50% of procedures in 2012 to 46% of procedures in 2022 (Figure 3). Office procedures grew from 14% to 21% of procedures over the same period. Off-campus outpatient hospitals began reporting procedures in 2016 and grew to 6% of all procedures by 2022. On-campus outpatient hospitals saw a decrease in usage from 36% to 26% of all procedures in the 10 years studied (Figure 3).

**Figure 4.**
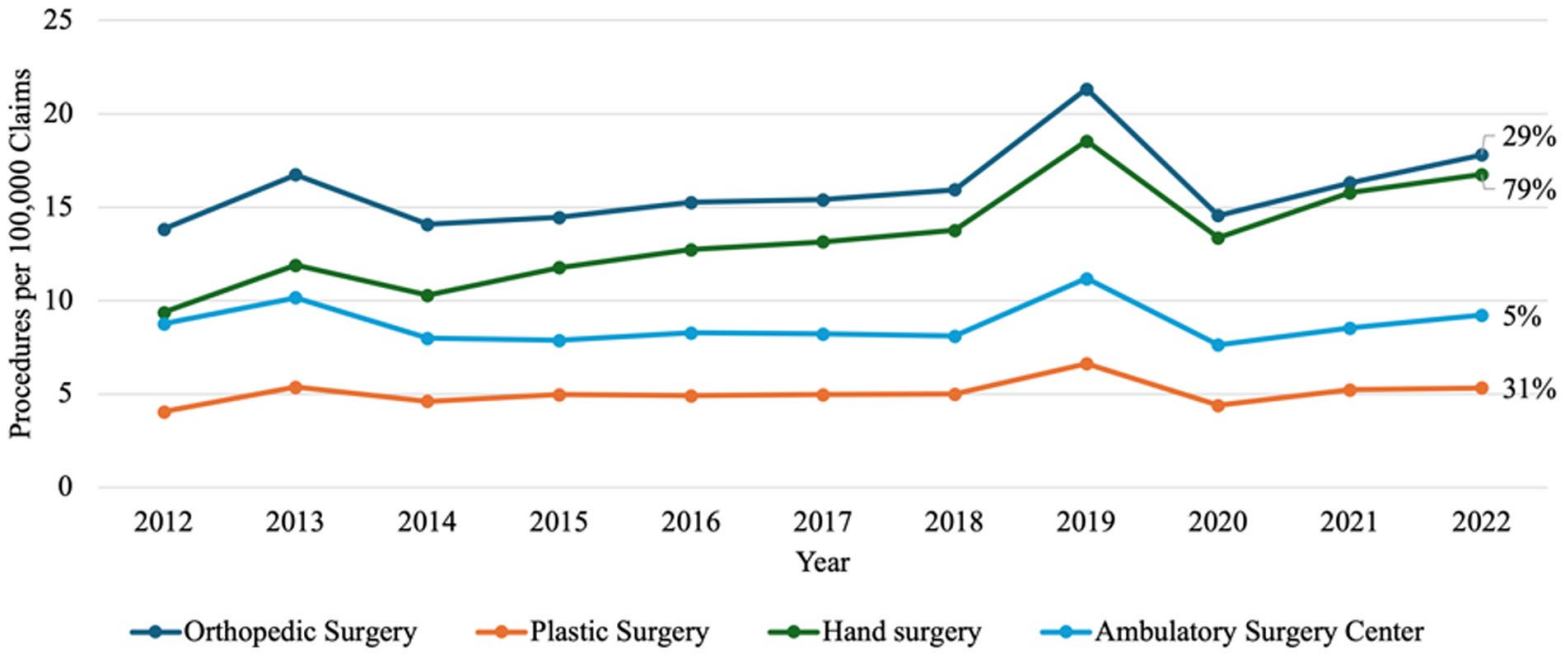
Provider for all procedures per 100 000 claims from 2012 to 2022: line graph showing annual rates of Dupuytren’s contracture procedures by provider specialty per 100 000 Medicare claims from 2012 to 2022. The y-axis represents procedure rates per 100 000 claims; the x-axis shows calendar year. Lines represent procedures performed by orthopedic surgeons, plastic surgeons, hand surgeons, and providers in ambulatory surgical centers.

Finally, when stratified by provider the 4 providers that met the requirement of performing more than 3% of procedures during the 10-year period, orthopedic surgeons emerged as the most common provider. Orthopedic surgeons performed 13.83 procedures per 100 000 claims in 2012 increasing to 17.81 in 2022 (Figure 4). The second most common were hand surgeons who had the largest increase at 79% from 9.73 to 16.75 over the 10 years (Figure 4). Plastic surgery increased 31% over 10 years from 4.05 to 5.33, while ambulatory surgery centers increased 5% from 8.78 to 9.21 per 100 000 claims (Figure 4).

## Discussion

In this retrospective observational analysis of 189 142 procedures in the Medicare and Medicaid patient population from 2012 to 2022, we have highlighted a general uptick in the utilization of our selected procedures for the treatment of DC, a dynamic change in the providers who are performing these procedures, and a shift in type of location of procedure. Importantly, a major goal of this study was to determine the impact of the 2020 removal of CCH for the treatment of DC in non-US markets on the frequency of its use within the United States. Predictably, previous studies have noted the sharp decline in the use of CCH following this decision.^5,6^ Our analysis taken from the Physician/Supplier Procedure Summary (PSPS) data hopes to provide clarity of the effects of this move in the United States.

Furthermore, in a broader sense, treatments for DC in the Medicare and Medicaid services have increased relative to the usage of the Medicare/Medicaid services (Figure 1). This could indicate that treatment has become more accessible in this patient population. However, previous studies have found conflicting trends compared to our treatment data that both confirm and contradict our findings. Gordon et al^17^ analyzed the treatment utilization trends and costs of various procedures for Dupuytren’s disease in the United States from 2012 to 2019 and found that the overall annual treatment rates for Dupuytren’s disease did not change significantly over time. Conversely, Jain et al^18^ reported a nearly 4-fold increase in the number of procedures to treat DC from 2015 to 2018, with open fasciectomy having the majority market share nationally and regionally. In addition, for more context on the treatment trends, socioeconomic factors certainly play a role in procedure utilization. Zhuang et al^19^ found that adverse socioeconomic determinants of health were associated with decreased treatment utilization for DC, indicating that patients with lower socioeconomic status, often covered by Medicaid, may have less access to certain treatments. Thus, it is promising to see that, though barriers may exist for the accessibility of DC treatments in the Medicare/Medicare population, they have not been to such an extent as to decrease the total treatment utilization in the population (Figure 1).

On a procedure-specific level, Jain et al^18^ also noted that needle fasciotomy was the least commonly performed procedure, despite being the least costly option. Our findings from the PSPS database indicate that open fasciotomy was the least performed procedure in the Medicare/Medicaid population, while needle fasciotomy was recorded at an intermediate level of frequency (Figure 1). Overall, open fasciotomy was the most common procedure in the Medicare/Medicaid population (Figure 1). These findings, for the most part, confirm previously recorded trends. Open fasciectomy remains the predominant treatment of DC, accounting for approximately 60.7% of procedures, and has shown an increase in the number of patients receiving release of one or more digits.^17^ Open fasciectomy has been favored due to its lower recurrence rates compared with other treatments, which could partially explain why this treatment constitutes the majority of included procedures.^20^ It is also the Dupuytren’s intervention favored in more severe forms of disease.^20-22^ The predominant use of this procedure in the Medicare/Medicaid population could possibly indicate limited access to early treatments, resulting in greater digit contractures requiring invasive treatment options. More investigation into the severity of DC in the Medicare/Medicaid population would highlight if this were truly the case.

Important to the goals of this paper, another notable trend in the frequency of treatments derived from Figure 1 is the huge increase in the utilization of CCH injections science 2012. Collagenase clostridium histolyticum injections work by enzymatic targeting and breaking down the collagen cords causing the contracture.^3^ This treatment modality is typically indicated for moderate contractures rather than more severe forms of the disease.^23,24^ CCH injections are sold under the name Xiaflex in the United States (Endo Pharmaceuticals Inc., Malvern, Pennsylvania) and Xiapex (Swedish Orphan Biovitrum AB, Stockholm, Sweden), are a minimally invasive option for DC with similar efficacy to percutaneous needle fasciotomy.^9,20,23,25^ As previously stated, the Xiapex, the non-US version of CCH injections were removed from non-US health care markets. This action has resulted in a marked decrease in the use of CCH outside of the United States.^5^ Previous inquiries, however, have not demonstrated if this has affected the US market. The data from the PSPS analyzed in this study indicate that this move *has not* affected CCH usage within the United States (Figure 1). Indeed, after the 2020 removal of Xiapex, Xiaflex usage within the United States increased by 25.7% from 2020 to 2022, confirming the strong growth of this treatment methodology in the Medicare/Medicaid population. Interestingly, the CCH utilization data in the PSPS contradicts a previous study by Gordon et al^17^ in 2023 which analyzed the PearlDiver nationwide administrative claims database and demonstrated relatively stagnant growth of most DC treatment options.

Understanding the DC treatments in the Medicare and Medicaid population also depends on the specific providers who perform the procedure. Orthopedic surgeons remain the most commonly recorded provider type for all 10 years of the selected time period (Figure 4). In addition to orthopedic specialists, hand specialists demonstrate a clear incremental increase in their share of the procedures, while plastic surgeons remain relatively stagnant in their growth (Figure 4). Judging by the data collected in this study, treatment for DC follows the increasing demand and utilization of hand specialists in the United States. Previous studies analyzing the demand and supply for hand surgery training in the United States from 2012 to 2023 showed a significant increase in the number of hand surgery programs and training positions, with a 27.4% increase in programs and a 28.7% increase in training positions.^26^ There are also several procedures that hand specialists are performing more frequently. For instance, the number of elective hand surgery operations rose by 34%, with carpal tunnel syndrome surgeries almost doubling and osteoarthritis-related surgeries nearly tripling.^27^ In addition, minor hand surgeries such as open carpal tunnel release, trigger digit release, and hand or finger mass excision are increasingly being performed in office-based procedure rooms rather than traditional operating rooms.^28^

Dupuytren’s contracture treatments are similar to the above hand conditions in terms of their surgical intensiveness, so it is no surprise that they are demonstrating similar trends. The data presented in Figure 3 highlights the growing pension for performing these procedures in more of an out- of- hospital setting. The causes behind this trend are multifactorial. First, as noted above, the more aggressive and invasive treatments, such as open fasciectomy, are growing less when compared with the rising prevalence of less invasive methods (Figure 1). Less invasive procedures thus need less intensive resources and are less likely to require the use of an operating room.^29^ Indeed, there is a growing trend among multiple minor hand surgery operations, ranging from carpal tunnel release, De Quervain release, and excision of hand masses.^28,30^ The transition from inpatient, or hospital-based procedure to outpatient or office-based procedures is driven by a variety of factors. Compared with an ambulatory surgery center, a minimally invasive hand procedure can be performed at a substantial cost reduction of up to 82%.^31^ In addition, performing procedures outside of operating rooms frees up time that could be dedicated to procedures that necessitate their use, producing additional benefit for patients and providers alike.^31^

A marked spike is visible across Figures 1, 2, and 4 for the year 2019, suggesting it may be an outlier in this dataset. A review of CPT coding corrections and policy changes issued by the American Medical Association between 2018 and 2020 revealed no modifications to the specific codes analyzed in this study (26121, 26123, 26125, 26045, 26040, 20527).^32-35^ It is difficult to reason a clear cause for this outlier. One possible explanation is that the 2019 data reflects a true increase in procedural volume for DC. However, the subsequent sharp decline in 2020 may be attributed to the onset of the COVID-19 pandemic, which significantly limited elective procedures worldwide, including those for nonemergent conditions like DC.^36,37^ The gradual rise in procedure rates from 2020 to 2022 may represent a postpandemic recovery phase, although by 2022, volumes had not yet returned to prepandemic (2019) levels. While this general phenomenon is well documented with other procedures, other possible reasons remain more difficult to prove. Additional considerations for this outlier could be administrative backlog or catch-up in processing procedural claims, delayed reporting, coding misclassifications, or an isolated increased incentive for the selected procedures in 2019. All these possibilities could, to some degree, explain the 2019 outlier, but no hard evidence is available to further support these alternative theories. For now, the authors’ best explanation for the spike in the values around 2019 would be difficulties in accessing non- emergency medical care during the COVID-19 Pandemic, distorting the overall trends.

Furthermore, the results and discussion of this retrospective observational study should be considered with its limitations in mind. First, our analysis requires that the coding for each procedure identification be completely accurate, as the results of our study depend on the veracity of the database information. Of course, with a database as large as the CMS PSPS, there are certainly some miscodings for procedure CPTs, as well as misclassifications of providers and the location of the procedure. Indeed, for the classification of providers, a more prominent issue arises with the fact that ambulatory surgery centers are listed as both a provider and a place of service. It is difficult to say what the true nature of the providers who were listed as ambulatory surgery centers for the purposes of this study and for other studies that employ a similar methodology. Furthermore, the patient information included within this study is limited to the Medicare and Medicaid populations. The trends in this study may not be reflected in the non-Medicare/Medicare population.

## Conclusion

This study demonstrates that the overall frequency of interventions for DC among the Medicare and Medicaid population has moderately increased over the past decade, with open fasciectomy remaining the dominant treatment modality. Despite its withdrawal from non-US markets, the use of CCH in the United States has continued to rise substantially, suggesting persistent confidence in its efficacy and a growing preference for less invasive treatment options. Orthopedic surgeons remain the leading providers of care for DC, although hand specialists have shown the most significant growth in procedural volume. In addition, there has been a noticeable shift toward performing these interventions in office-based and ambulatory surgical settings, reflecting broader trends toward cost-effective, outpatient management of hand conditions. These findings highlight evolving treatment preferences and health care delivery models for DC in the United States, reinforcing the importance of continued monitoring of practice patterns and access to emerging therapies.
